# Potato Zebra Chip: An Overview of the Disease, Control Strategies, and Prospects

**DOI:** 10.3389/fmicb.2021.700663

**Published:** 2021-07-22

**Authors:** Victoria Mora, Manikandan Ramasamy, Mona B. Damaj, Sonia Irigoyen, Veronica Ancona, Freddy Ibanez, Carlos A. Avila, Kranthi K. Mandadi

**Affiliations:** ^1^Texas A&M AgriLife Research and Extension Center, Weslaco, TX, United States; ^2^Department of Agriculture, Agribusiness, and Environmental Sciences, Citrus Center, Texas A&M University-Kingsville, Weslaco, TX, United States; ^3^Department of Entomology, Minnie Bell Heep Center, Texas A&M University, College Station, TX, United States; ^4^Department of Horticultural Sciences, Texas A&M University, College Station, TX, United States; ^5^Department of Plant Pathology and Microbiology, Texas A&M University, College Station, TX, United States

**Keywords:** Fastidious bacteria, zebra chip, psyllids, *Candidatus* Liberibacter solanacearum, *Solanaceae*, Resistant varieties, crop improvement

## Abstract

Potato (*Solanum tuberosum* L.) is an important food crop worldwide. As the demand for fresh and processed potato products is increasing globally, there is a need to manage and control devastating diseases such as zebra chip (ZC). ZC disease causes major yield losses in many potato-growing regions and is associated with the fastidious, phloem-limited bacterium *Candidatus* Liberibacter solanacearum (*C*Lso) that is vectored by the potato-tomato psyllid (*Bactericera cockerelli* Šulc). Current management measures for ZC disease mainly focus on chemical control and integrated pest management strategies of the psyllid vector to limit the spread of *C*Lso, however, they add to the costs of potato production. Identification and deployment of *C*Lso and/or the psyllid resistant cultivars, in combination with integrated pest management, may provide a sustainable long-term strategy to control ZC. In this review, we provide a brief overview of the ZC disease, epidemiology, current management strategies, and potential new approaches to manage ZC disease in the future.

## Introduction

Potatoes (*Solanum tuberosum* L.) constitute a centuries-old world dietary staple, with total world production estimated at 368.2 million tons in 2018 (Faostat, 2020). The United States is the fifth largest potato producer, after China, India, Russia, and Ukraine (Faostat, 2020), with an industry valued at ∼3.5 billion (USDA, 2019; Faostat, 2020). About one-third of United States grown potatoes are for processing, of which 63–83% are for frying, chipping and other packaged products, and the rest for fresh market, fodder, or used as seed (USDA, 2019). Potato domestication resulted in cultivars with reduced glycoalkaloid tuber content, making them more palatable and leading to increased tuber size and improved carbon fixation and transport (Spooner et al., 2014; Machida-Hirano, 2015). Few hardy wild potatoes were also crossed with their cultivated relatives to improve disease resistance, yield and quality for almost a century (Jansky et al., 2013). This yielded highly marketable improvements, like enhanced processing quality for chipping and frying, and resistance to some viruses and nematodes (Douches et al., 1996; Hirsch et al., 2013; Bethke et al., 2017). However, their low genetic diversity led to vulnerability to pests and diseases, and acute inbreeding depression.

## Early Reports of Zebra Chip Disease

**FIGURE 1:**
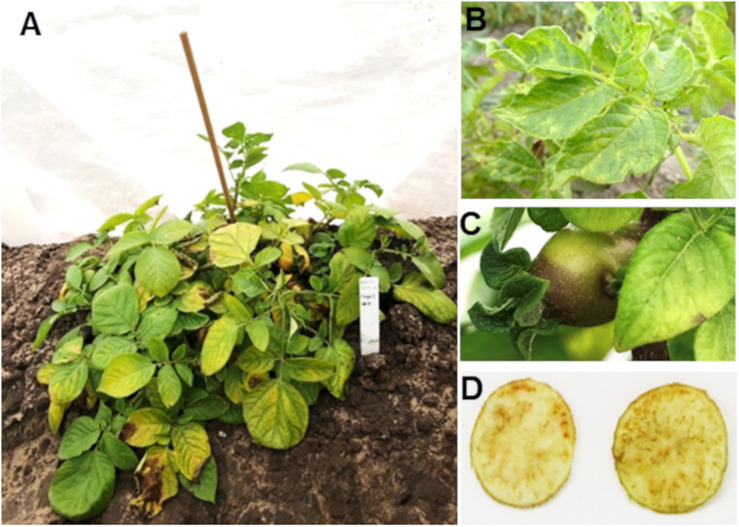
Characteristic symptoms of zebra chip (ZC) disease. Infection of *Candidatus* Liberibacter solanacearum (*C*Lso) often results in **(A,B)** chlorosis and upward curling/rolling of leaves, stunted plants, **(C)** aerial tuber growth, and **(D)** necrotic flecking/browning of tubers/chips and overall reduction of marketable yield.

Zebra chip (ZC) disease was first reported in 1994 in Saltillo, Mexico, and later in South Texas, United States in 2000 (Munyaneza et al., 2007, 2009), The fastidious phloem-limited bacterium, *Candidatus* Liberibacter solanacearum (*C*Lso), was identified as a putative causal agent. *C*Lso is transmitted to plants by the potato-tomato psyllid *Bactericera cockerelli* Šulc (Munyaneza et al., 2007; Hansen et al., 2008; Liefting et al., 2009). Vegetative symptoms of ZC disease on plants include leaf chlorosis, discoloration, curling or upward rolling, aerial tubers, axillary bud proliferation, stunted growth, and eventually premature plant death (Figure 1). *C*Lso-infected potato tubers are often deformed and of poor quality, exhibiting collapsed stolons, vascular ring browning and brown flecks. When fried for chipping, the brown discoloration becomes darker, making chips bitter to taste, and unmarketable (Figure 1D; Secor and Rivera-Varas, 2004). Beyond North America, ZC disease is also documented in South America, New Zealand, and Australia (Hansen et al., 2008; Liefting et al., 2008a, 2009; Teulon et al., 2009; Crosslin et al., 2012; Munyaneza, 2012; Vereijssen et al., 2018).

Despite the relatively recent origins of ZC, potato psyllid infestation was first documented in peppers in Colorado, United States and was described as a potential pest in 1909 by Šulc (1909). The detrimental effects of psyllids were not fully noticed until 1927, when vast outbreaks of what was then described as psyllid yellows (PY) disease led to reduction of potato yields in Utah to the Rocky Mountain states of the United States (Linford, 1928; Richards, 1928). The description of the PY foliar symptoms (Arslan et al., 1985) was very similar to the foliar symptoms of ZC (Pitman et al., 2011; Figure 1). Although initially PY was thought to be associated with toxins released by psyllid feeding, so far, no other pathogens nor toxins have been associated with PY. Hence it led to a hypothesis that PY could be a mild case of ZC, wherein *C*Lso was present at low, undetectable levels in the affected plants (Richards and Blood, 1933; Carter, 1939; Arslan et al., 1985; Munyaneza et al., 2011; Monger and Jeffries, 2018).

Nevertheless today, the potato psyllid is considered an A1 quarantine pest by the EPPO (European and Mediterranean Plant Protection Organization), and as a primary vector for *C*Lso, together cause significant economic losses (PM, 2017).

## *C*Lso-Potato Psyllid Host Range, Transmission, and Diagnostics

In addition to causing ZC disease on potatoes, *C*Lso can be transmitted to and infect other solanaceous crops such as tomato (*S. lycopersicum*), tomatillo (*Physalis* spp.), eggplant (*S. melongena*), pepper (*Capsicum* spp.), tobacco (*Nicotiana tabacum*), and tamarillo (*Solanum betaceum*; Hansen et al., 2008; Liefting et al., 2008b, 2009; Munyaneza et al., 2009, 2013, 2014; Aguilar et al., 2013). *B. cockerelli* is the main *C*Lso vector to infect these solanaceous crops in Mexico, United States, Central America (Guatemala, Honduras, and Nicaragua), Ecuador, Canada, New Zealand, and Australia (Liefting et al., 2008a; Munyaneza et al., 2009; Bextine et al., 2013; Thomas et al., 2018; Carrillo et al., 2019; Henrickson et al., 2019). Few wild solanaceous species can serve as a reservoir for both *B. cockerelli* and *C*Lso (Henne et al., 2010; Murphy et al., 2014; Vereijssen et al., 2015). Studies have found certain psyllid haplotypes (Northwestern Haplotype) can overwinter on natural vegetations such as bittersweet nightshade (*Solanum dulcamara* L.; Murphy et al., 2013, 2014; Horton et al., 2015) and can remerge in the Summer to infect agronomic crops. Similarly, in New Zealand, both *C*Lso and *B. cockerelli* were found on bittersweet nightshade and thorn-apple (*Datura stramonium*; Vereijssen et al., 2015). Further studies to determine specific *C*Lso haplotypes prevalent in the wild species and weedy plants will provide new insights into the significance of reservoir hosts in *C*Lso and ZC epidemiology (Bradshaw and Ramsay, 2005).

Feeding on infected plants is the main mode of *C*Lso acquisition by adult psyllids and nymphs (Buchman et al., 2011). After acquisition, there is a 2-week latent period before the infected psyllid is able to transmit the bacterium into new plant tissues (Sengoda et al., 2013). Upon feeding on a plant, it takes as little as 1 h for *C*Lso to be transmitted into plant tissues (Buchman et al., 2011). Subsequently, depending on the host plant, it can take approximately 3 weeks for the onset of ZC symptoms (Charkowski et al., 2020). Within an infected plant, *C*Lso is not evenly distributed and as such is present in low levels (Charkowski et al., 2020). Polymerase chain reaction (PCR) and/or quantitative PCR is the most widely used diagnostic approach for detecting *C*Lso in both the host plants and the psyllids, and can be used to distinguish the different haplotypes (Hansen et al., 2008; Secor et al., 2009; Swisher et al., 2012; Ananthakrishnan et al., 2013; Beard and Scott, 2013; Beard et al., 2013; Contreras-Rendón et al., 2020). Other emerging technologies such as Raman Spectroscopy are also being explored to detect ZC disease, that allows for rapid, non-invasive and in-field diagnostics (Farber et al., 2021).

## *C*Lso Haplotypes and Diversity

Twelve different *C*Lso haplotypes have been reported so far [A, B, C, D, F, G, H, H (Con), U, Cras1 and Cras2] (Wen et al., 2009; Munyaneza et al., 2010; Nelson et al., 2011, 2013; Teresani et al., 2014; Haapalainen et al., 2018, 2020; Mauck et al., 2019; Swisher Grimm and Garczynski, 2019; Contreras-Rendón et al., 2020; Sumner-Kalkun et al., 2020). In addition to *B. cockerelli*, other relatives in the Triozidae family (Hemiptera) transmit certain *C*Lso haplotypes. For example, haplotype C found in carrots is vectored by *Trioza apicalis* Förster (Munyaneza et al., 2010). Haplotypes D and E are transmitted by the carrot psyllid vector, *Bactericera trigonica* Hodkinson (Nelson et al., 2011; Swisher et al., 2014; Borges et al., 2017; Charkowski et al., 2020). While, *C*Lso haplotype U identified in northern Europe, is associated to *Trioza urticae* psyllid (Haapalainen et al., 2018). In the Americas, ZC disease is primarily associated with the haplotypes A, B, and F. *C*Lso A and B are transmitted by *B. cockerelli*, while the vector of haplotype F is still unknown (Hansen et al., 2008; Wen et al., 2009; Nelson et al., 2011; Swisher Grimm and Garczynski, 2019). In New Zealand and Norfolk Island (Australia) the *C*Lso haplotype A vectored by *B. cockerelli* interaction is considered the predominant haplotype causing ZC disease (Liefting et al., 2008a; Nelson et al., 2011; Thomas et al., 2018). Taken together, *C*Lso haplotypes A and B appear to be the most predominant across the world, in the Americas, New Zealand, and Australia, and associated with the ZC disease in potatoes (Rosson et al., 2006; Liefting et al., 2008a; Nelson et al., 2011; Thomas et al., 2018; Savary et al., 2019; Delgado et al., 2020).

Studies with *C*Lso haplotypes A and B showed that both haplotypes can infect plants either individually, or as co-infections (Harrison et al., 2019). Haplotype distribution and resulting effects on disease severity in single or co-infections were also studied in tomatoes and potatoes (Mendoza-Herrera et al., 2018; Harrison et al., 2019). For instance, infection of haplotype B is detrimental to tomato plants, as they usually die before fruit development, whereas plants can remain alive with symptoms when infected with haplotype A (Mendoza-Herrera et al., 2018). In potatoes, haplotype B induces greater ZC symptoms in tubers than haplotype A (Grimm et al., 2018), and dual-haplotype AB infections usually result in greater severe symptoms than infections with only haplotype B (Hernández-Deheza et al., 2018; Harrison et al., 2019). Interestingly, haplotype B seems to lower psyllid nymph survival rate, compared to those carrying haplotype A (Yao et al., 2016).

## ZC Control: Psyllid Monitoring, Chemical, Biological and Integrated Pest Management

Currently, a primary approach to manage ZC is by controlling the psyllid vector populations. Components of integrated pest management (IPM) such as chemical, cultural, and biocontrol strategies have been implemented worldwide (Vereijssen et al., 2018). Extensive monitoring and detection of psyllid population are also being used to determine psyllid movements (Butler and Trumble, 2012). Data gathered from monitoring psyllids on sweep nets are correlated with psyllid-vectored diseases in tomato fields (Pletsch, 1947; Cranshaw, 1994). Generally, psyllid infestations start along the perimeter of a field, moving toward the center as their population increases (Wallis, 1955; Cranshaw, 1994). Evidence of psyllid infestation can also be obtained by leaf examination, though tedious and time consuming (Pletsch, 1947; Goolsby et al., 2007). While, other studies have found sticky traps to be useful for monitoring psyllid populations, even at low densities (Goolsby et al., 2007).

For psyllid control, pesticide use has been the main course of action in several regions. Typical pest management guidelines for potato psyllids include the application of neonicotinoids like imidacloprid and thiamethoxam at planting as a seed treatment, with a subsequent foliar application to control adults and nymphs (Prager et al., 2013; Vereijssen et al., 2015; Nuñez et al., 2019). Unfortunately, excessive use of pesticides led to incidences of neonicotinoid resistance in Southwestern United States, South Texas, and Northern Mexico (Prager et al., 2013; Chávez et al., 2015; Szczepaniec et al., 2019). As such pesticide reliance is both economically and environmentally unsustainable.

Some cultural methods for the control of psyllids have also been tested. Such as by using certified clean seed, and planting non-host plants in crop rotations to maintain disease free planting areas (Vereijssen et al., 2018). In warmer climates such as in Southern United States, planting dates could be altered to delay exposure to potato psyllids (Guenthner et al., 2012). Few organic farmers have also found some success using physical barriers such as mesh covers to lower psyllid infestations (Merfield et al., 2015).

Lastly, biocontrol strategies have also been employed. Natural enemies of the psyllid, such as ectoparasitoids, coccinellids, and entomopathogenic fungi have shown promising effects against psyllids, by parasitizing them at multiple life stages, in greenhouse and laboratory studies (Al-Jabr, 1999; MacDonald et al., 2010; Lacey et al., 2011; Walker et al., 2011; Mauchline and Stannard, 2013; Rojas et al., 2015). Deployment of such natural enemies as biocontrol agents in greenhouse production systems (e.g., tomato) or in the field-scale (e.g., potato) could allow growing an earlier crop and reduce reliance on insecticides.

## Host Plant Resistance and Breeding Strategies for ZC Resistance

Efforts were made to study host plant resistance toward developing ZC resistant potato cultivars. Plants employ different mechanisms to protect themselves against pathogens and insects. Some host-plant resistance mechanisms are constitutive, such as physical or pre-formed structural barriers and release of chemicals that disrupt pathogen transmission, insect feeding, and oviposition. Other plant defenses, such as volatile compounds emission or upregulation of resistance genes can also be triggered in response to a pest or pathogen (Dicke and Van Poecke, 2002; War et al., 2012). The host resistance mechanisms to pests can also be categorized as antixenosis and antibiosis. Generally, antixenosis refers to a deterring effect that plants can have on insect behavior, where antibiosis affects their lifecycle and reproduction (Painter, 1951; Kogan and Ortman, 1978; Smith, 2005).

In the case of ZC, several varieties of potato and potato hybrids were identified to possess some degree of tolerance to ZC disease. In some varieties, tolerance was attributed to the antixenotic effects of glandular trichomes (Butler et al., 2011; Diaz-Montano et al., 2014; Rubio-Covarrubias et al., 2017). While few varieties appear to have a genetic basis for tolerance to *C*Lso in addition to having effects on the psyllid behavior (Rashidi et al., 2017; Fife et al., 2020). Recently, few wild-relatives of tomato, *S. pennelli*, and *S. corneliomulleri* were identified to possess resistance to *B. cockerelli* (Avila et al., 2019), with several quantitative trait loci (QTL) associated with insect mortality and lower fecundity in *S. habrochaites*. Such QTL in wild species could be a valuable source for breeding resistance to cultivars, however, their complex inheritance, modes of action, and pathogen-vector-host interactions require further characterization.

## Future Prospects and Strategies for ZC Resistance

In the past, lack of advanced genomic tools, combined with the cost effectiveness of chemical control strategies led to heavy reliance on pesticides, rather than prioritizing the development of new resistance varieties to pests/pathogens (Rowe, 1992; Spooner and Bamberg, 1994). However, recent advances in genomics and genetics resources (Varshney et al., 2005; Broekgaarden et al., 2011) including those for potato^1^, should help in identifying desirable traits, alleles, and marker development to develop new ZC resistance cultivars. For instance, the availability of the potato reference genome sequence, the discovery of SNPs in elite North American potato germplasm and the development of the Infinium 8,303 potato array have helped in identification of genes linked to improved agronomic traits (Hamilton et al., 2011; Massa et al., 2011; Felcher et al., 2012). The resources also enabled marker-assisted selection (MAS), which helps identify markers tightly linked to a target locus, instead of relying on phenotypic selection alone in making selections for crosses. Thus, MAS can be used to accelerate introgression of desirable ZC tolerance traits from various potato breeding clones or wild species into cultivar development. Several studies showed the potential of improving potato traits by increasing heterozygosity and genetic diversity of parental clones (Mendoza and Haynes, 1974; Bradshaw and Ramsay, 2005; Jansky and Peloquin, 2006). Thus, more focus will need to be given for identification and introgression of alleles from a diverse pool of genetic resources, including wild species, landraces, and cultivated potatoes (Bethke et al., 2019).

Introgression of desirable traits from related or distant species to cultivated potatoes using genetic engineering (GE) can be a viable alternative to speed cultivar development and reduce introgression of undesirable genetic material or traits (Halterman et al., 2016). Few example, GE potatoes that received United States regulatory approval include the “NewLeaf” Bt potatoes for resistance against Colorado beetle (*Leptinotarsa decemlineata*), “Innate^TM^’’ potatoes with resistance to fungal disease (late blight) and acrylamide formation^2^ (Halterman et al., 2016). Despite the significant advantages of GE crops, the costs associated with R&D and regulatory approval is tremendous and necessitates private sector investments, or public-private partnership. Furthermore, the GE products face marketing hurdles due to public skepticism (Halterman et al., 2016).

Selected traits can also be modified/introduced by genome editing technologies such as TALEN or CRISPR-Cas9 without introducing new foreign DNA (Wolt et al., 2016; Hameed et al., 2018). Derived plant products potentially face less regulatory scrutiny and approval burden. For instance, the United States regulatory body (USDA APHIS) determined that several transgene-free, genome-edited potato plants with disease resistance and other superior agronomic traits, would not be considered regulated under 7 CFR part 340 (Wolt et al., 2016). Although this does not preclude regulation by other agencies world-wide, it is nevertheless a significant advantage when it comes to commercialization.

## Conclusion

Since its first report in 1994, ZC disease is now established in several potato producing regions worldwide. The putative causal agent, *C*Lso, can also infect other economically significant *Solanaceae* crops, thus posing an even more threat to the agricultural industry. IPM strategies (chemical, cultural, and biological control) have been implemented to manage psyllid vector population and limit ZC disease. However, we still need long-term solutions. Recent developments in potato genetic resources and crop improvement technologies could be further leveraged for developing new potato cultivars with genetic resistance to the psyllid and/or *C*Lso. In combination with IPM practices, the ZC resistant or tolerant cultivars could be deployed in the future to effectively manage ZC disease.

## Author Contributions

KM supervised the study. All others contributed to the preparation and editing of the review.

## Conflict of Interest

The authors declare that the research was conducted in the absence of any commercial or financial relationships that could be construed as a potential conflict of interest.

## Footnotes

1 http://solcap.msu.edu/; https://www.polyploids.org/

2 https://apnews.com/article/1d3c790ad18f4e828598ccf44ec047bf
